# Comprehensive Survey of the Litter Bacterial Communities in Commercial Turkey Farms

**DOI:** 10.3389/fvets.2020.596933

**Published:** 2020-12-04

**Authors:** Bishnu Adhikari, Guillermo Tellez-Isaias, Tieshan Jiang, Brian Wooming, Young Min Kwon

**Affiliations:** ^1^Department of Poultry Science, University of Arkansas, Fayetteville, AR, United States; ^2^Cargill, Inc., Minneapolis, MN, United States; ^3^Cell and Molecular Biology Program, University of Arkansas, Fayetteville, AR, United States

**Keywords:** turkey, commercial farms, litter microbiotas, 16S rRNA gene sequencing, *Clostridium septicum*

## Abstract

The importance of microbiota in the health and diseases of farm animals has been well-documented for diverse animal species. However, studies on microbiotas in turkey and turkey farms are relatively limited as compared to other farm animal species. In this study, we performed a comprehensive survey of the litter microbiotas in 5 commercial turkey farms in the Northwest Arkansas (H, M, V, K, and R farms) including one farm with positive incidence of cellulitis (R farm). Altogether 246 boot swabs were used for 16S rRNA gene profiling of bacterial communities. At phylum level, 11 major bacterial phyla (≥0.01%) were recovered. At genus level, 13 major bacterial genera were found whose relative abundance were ≥2%. The microbial composition at both phylum and genus levels as well as their diversities varied across different farms, which were further affected by different flocks within the same farms and the ages of turkeys. Generally, the Firmicutes were higher in the flocks of younger birds, while the Actinobacteria and Bacteroidetes were higher in the flocks of the older birds. The Proteobacteria were highly enriched (47.97%) in K farm housing 56-day-old turkeys (K-56), but Bacteroidetes were found the highest in the flock C of M farm housing 63-day-old turkeys (M-C-63; 22.38%), followed by K-84 group (17.26%). Four core bacterial genera (*Staphylococcus, Brevibacterium, Brachybacterium*, and *Lactobacillus*) were identified in all samples except for those from R farm. In contrast, 24 core bacterial genera were found based in all cellulitis-associated samples (R farm), including *Corynebacterium*, an unknown genus of family Bacillaceae, *Clostridium* sensu stricto 1 (>97% similarity with *C*. *septicum*), and *Ignatzschineria* among others, suggesting their possible roles in etiopathogenesis of cellulitis in turkeys. Overall results of this study may provide valuable foundation for future studies focusing on the role of microbiota in the health and diseases of turkeys.

## Introduction

During the last decade, the decrease in sequencing costs coupled with innovations in computational technologies (1) has remarkably advanced our understanding of the composition and function of microbial communities residing in diverse environments (2). Accordingly, the roles of microbiota in health and diseases have been well-documented in wide range of animals, yet limited microbiota studies have been conducted so far in turkeys.

One study investigated the succession of intestinal microbiota in the ceca of male turkeys, where decrease in *Clostridium* species and increase in *Bacteroides uniformis* were reported over time (3). The cecal bacterial succession in relation to the *Campylobacter jejuni* and *Campylobacter coli* loads has also previously been reported (4). Similar with the previous findings, the cecal bacterial communities were changed in a time-dependent manner and *Campylobacter* loads were correlated with the acute microbial community transition. In another study, considerable divergence of the cecal bacterial genera was found in the domestic turkeys as compared to the wild ones, though bacterial compositions at higher taxonomic levels were similar (5). Although, these studies provide valuable insights regarding intestinal microbiota in turkeys, they are based on low-resolution molecular fingerprinting methods, such as terminal restriction fragment length polymorphism (T-RFLP) or automated ribosomal intergenic spacer analysis (ARISA) (3–5). These methods have certain limitations in terms of accurately depicting microbial diversity in samples, especially for those samples with higher level taxon richness (6).

Along with the advancement in sequencing technologies, the intestinal microbiota of turkey has been investigated using high-throughput next generation sequencing of 16S rRNA genes (7–10). These studies were conducted in turkeys to characterize the microbiota along the gastrointestinal tract (10), litter microbiotas (8), and their relation in terms of body weight gain (7), antibiotics treatment (8), and hemorrhagic enteritis virus (9). Mostly, these studies were conducted in experimental animal settings which might not properly reflects the turkey microbiotas in commercial farms, demanding the need of more comprehensive survey of turkey microbiota in commercial farms.

In this study, we characterized the litter microbiotas from different flocks of five different commercial farms at different time points of turkey production. We used the boot swab samples for better representation of microbiotas present at the farm level. A previous study demonstrated that the litter microbiotas in turkey were most closely related to the ileal microbiotas among other regions (8), suggesting the litter microbiota data in this study might reflect well the dynamic changes in the ileal microbial communities of turkeys in the respective farms.

## Materials and Methods

### Collection of Samples

Samples were collected from five commercial turkey farms (H, M, V, K, and R) in the Northwest Arkansas at different time points including one farm (R) that had incidence of cellulitis at the time of the sampling. All these farms were individually owned and operated under the contract of Cargill, Inc during the period of the sampling. From all farms except for R farm, the samples were collected from each side of the barn's quadrant by walking with a pair of boots with sponge swab attached at the bottom. Since each barn has four quadrants, a total of 8 (4 × 2 = 8) samples were collected from each barn. From R farm, samples were collected to be used as a cellulitis-positive samples. That is, four sponge swab samples directly from the birds with cellulitis (R farm Bird: RB) and four boot sponge swab samples from the litter surrounding the cellulitis-positive birds (R farm Litter: RL) were collected. Total 246 sponge swab samples were collected and used for 16S rRNA gene profiling analysis. The summary of the samples with the information on the farms, flocks, age of birds, and number of samples is shown in Table 1.

**Table 1:** Summary of the farm samples used for microbiota analysis.

**Farm**	**Incidence of cellulitis**	**Flock**	**Age (days)**	**Swab sample type**	**No. of samples**
H	Unknown	A	33	Litter	8
			84		16
			105		16
		B	49		8
			70		16
			103		16
M	Unknown	A	84	Litter	16
		B	98		16
		C	28		8
			63		16
			98		16
V	Unknown	A	58	Litter	8
			112		14
		B	59		8
			80		16
			115		16
K	Unknown		28	Litter	8
			56		8
			84		8
R	Positive		60	Litter (RL): 4	8
				Bird (RB): 4	
Total					246

### DNA Extraction

We developed the protocol for efficient extraction of metagenomic DNA in boot swab samples. For this purpose, each sponge swab sample was transferred to the sterile stomacher bag with filter (Seward). After adding 20 ml of sterile PBS buffer, the sponge swab samples were stomached for 2 min in a stomacher (Lab Blender 400 series). In order to obtain uniformity in sponge samples, litter debris attached to each samples were removed aseptically before transferring to stomacher bags. The filtered contents from each sample after stomaching were transferred to 15 ml sterile tube and centrifuged @8,000 rpm for 10 min to make cell pellets. The supernatant from each sample after centrifugation was removed, whereas pellets containing bacterial cells were retained and used for DNA extraction using QIAamp Fast DNA Stool Minikit (Qiagen). All the procedures for DNA extraction were followed according to the manufacturer's instructions except for the incorporation of a bead beating step. Bead beating step was incorporated in the protocol because bead beating was reported to increase DNA yield and taxon abundances (11). For bead beating, pellet from each sample was resuspended in 1 ml Inhibit Ex Buffer provided with the kit, which was then transferred to 2 ml microcentrifuge tube with a screw cap (Thermofisher Scientific) containing 0.25 ml of sterile 0.1 mm glass leads (BioSpec). Bead beating was performed using Bead mill 24 (Fisher Scientific) for 6 cycles where 0.30 s run for each cycle and 0.11 s stopping time between the cycles. After bead beating, samples were incubated at 70°C for 10 min, followed by manufacturer's protocol for downstream steps and finally DNA was eluted in 30 μl of elution buffer. Negative control sample was also included in the DNA extraction step.

### PCR and Library Preparation for Sequencing

V4 region of 16S rRNA gene from genomic DNA of each farm sample and a negative control from DNA extraction step and a positive control using a mock community DNA sample (ZymoBIOMICS Microbial Community DNA Standard II, Zymo Research, Cat# D6311) was amplified using the primers 515F (12) and 806R (13). The library of amplicons for sequencing was prepared according to the 16S Illumina PCR protocol described in the Earth Microbiome project [http://www.earthmicrobiome.org; (14)] with slight modifications. In brief, Platinum™ II Hot-Start Green PCR Master Mix (2X) user guide protocol (Thermofisher Scientific, Catalog No. 14000013) was used to conduct PCR in a 25 μl final reaction volume and 35 amplification cycles. The thermocycling condition of PCR included an initial denaturation step at 94°C for 2 min, followed by 35 cycles of 0.5 min at 94°C, 0.5 min at 60°C, and 0.5 min at 68°C, and a final extension of 5 min at 68°C. The length of amplified product was confirmed with 1% agarose gel electrophoresis and equal amount (~300 ng) of amplicons from each sample as measured by Qubit dsDNA BR Assay Kit (ThermoFisher Scientific, Catalog No. Q32850) were pooled together. The pooled amplicons were finally ran on 1% agarose gel electrophoresis, purified using Zymoclean Gel DNA Recovery Kit (Zymo Research, Catalog No. D4007), and sequenced using Illumina MiSeq with paired end 300 cycle options.

### Amplicons Sequence Analysis

Nebula cloud computing platform at the University of Arkansas was used to process raw sequencing reads in QIIME 2 version 2018.8 (15) utilizing the pipelines developed for paired-end data types. In sum, “demux emp-paired” method of q2-demux plugin was used to demultiplex sequencing reads followed by quality filtering and denoising with “dada2 denoise-paired” method of q2-dada2 (16) plugin available at QIIME 2. The truncation length of forward and reverse reads was set at 240 and 200 bp, respectively, which is based on the quality score criteria (≥30). Taxonomic assignment was performed using a Naive Bayes classifier (17) pre trained with SILVA (Version 132) 99% OTUs (18, 19) and q2-feature-classifier plugin, where the sequences have been trimmed to include only the V4 region of the 16S rRNA gene defined by the 515F/806R primer pair. The core-metrics-phylogenetic method at a sampling depth of 17,000 was used to analyze Alpha and Beta diversity. Alpha diversity calculated by Shannon's diversity index (20) and Observed OTUs metric, while beta diversity calculated by unweighted UniFrac distance metric (21) and Bray Curtis (22) are presented. All figures except Emperor plots were created using ggplot2 packages of R (23). The significant differences in alpha diversity were calculated using alpha-group-significance command of QIIME2 which uses Kruskal-Wallis test. In contrary, statistical differences in beta diversity among groups were calculated by PERMANOVA (24) test using beta-group-significance command of QIIME2 with pairwise option. For both diversities analysis, the corrected *P*-values for multiple comparisons (*q*) were used to report significant difference between two groups, where the level of significance was set at adjusted *P* < 0.05.

## Results

### Summary of DNA Sequencing Analysis

The summarized feature table resulted in total 10,863,650 sequence reads from the 246 samples that ranged from 17,134 to 82,383 reads per sample. The median and mean ± SE reads per sample were 42,949.5 and 44,161.2 ± 787.9, respectively. In addition, there were altogether 3,057 unique features (ASV) from all 246 samples.

### Phylum Level Compositions of Litter Bacterial Communities

**Figure 1:**
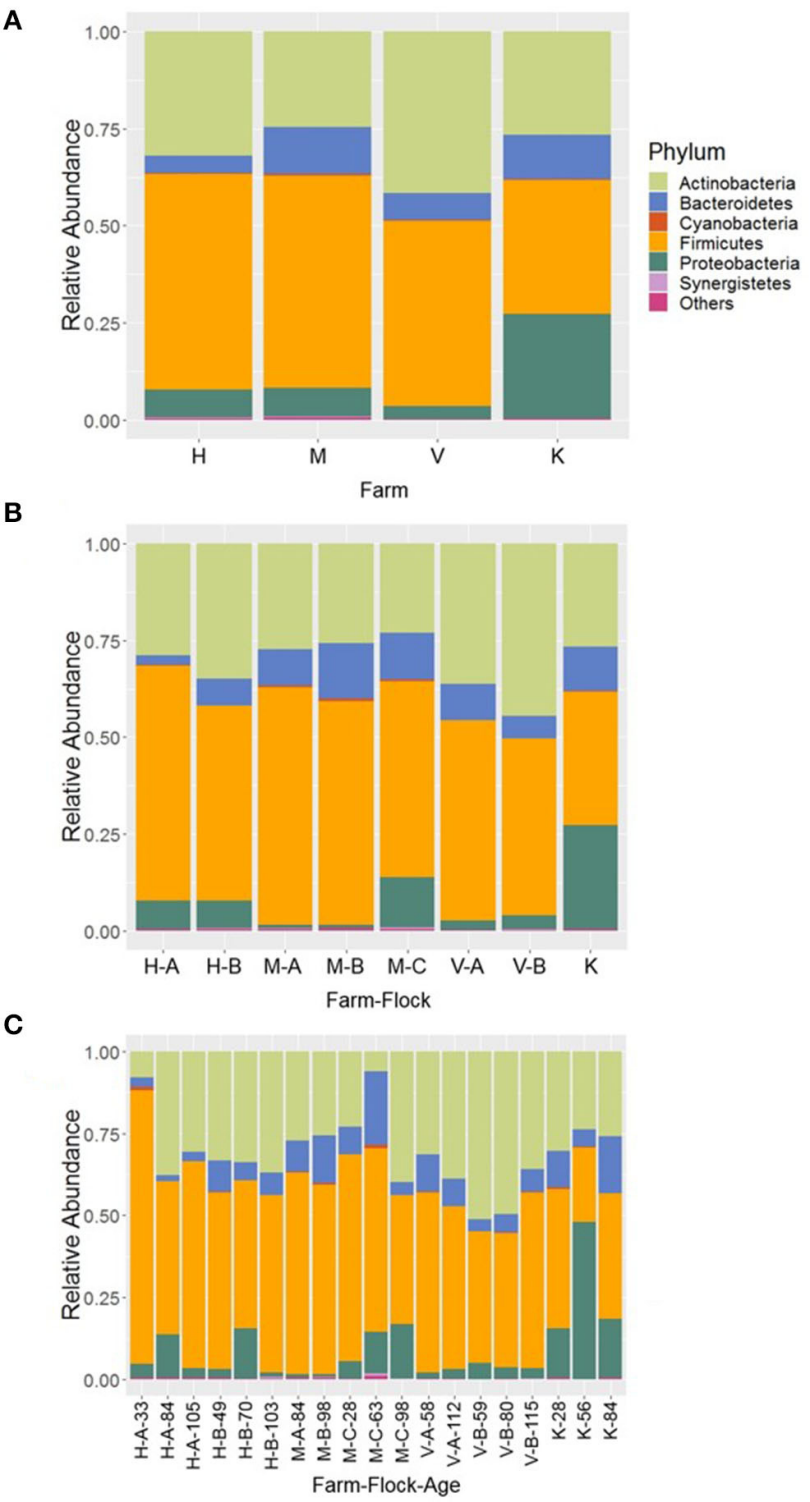
Composition of the litter microbiotas in four different commercial turkey farms of Northwest Arkansas at phylum level for different **(A)** farms, **(B)** farm-flocks, and **(C)** farm-flock-ages. “Others” represent the minor phyla whose relative abundance were <0.1%.

At phylum level, 11 major bacterial phyla and one phylum (Euryarchaeota; 0.08%) that belongs to the domain Archaea were detected from four farm samples, excluding the samples from R farm (cellulitis-positive farm). These phyla constituted 99.96% of the total sequence reads. Among the major bacterial phyla, Firmicutes was the predominant phylum (51.10%), followed by Actinobacteria (31.69%), Proteobacteria (8.30%), and Bacteroidetes (8.18%). Other minor phyla included Cyanobacteria, Synergistetes, Epsilonobacteraeota, Kiritimatiellaeota, Tenericutes, Fusobacteria, and Verrucomicrobia whose relative abundance ranged from 0.01 to 0.24% and constituted <1% in total. The relative abundance of the major phyla across four different farms is shown in Figure 1A. Irrespective of farms, the Firmicutes was the predominant phylum which was found the highest in H farm (55.47%), while it was the lowest in K farm (34.49%) as shown in Figure 1A. On the contrary, Proteobacteria was found the highest in K farm (26.92%), whereas the Actinobacteria was found the highest in V farm (41.51%). The phylum Bacteroidetes was found the highest in M farm (12.04%) as shown in Figure 1A.

In addition, the microbial compositions at phylum level were also different among the flocks within the same farm as illustrated in Figure 1B. The variations in the relative abundance of major phyla among the flocks of same farm was further achieved due to differences in the ages of birds as illustrated in Figure 1C. Generally, Firmicutes was found higher in each flock of the farms rearing younger birds, while the Actinobacteria and Bacteroidetes were found higher in the flocks of the older birds (Figure 1C). However, their relative abundance varied depending upon the farms and flocks within the same farm, but does not show apparent patterns of change over the ages. Similarly, the Proteobacteria was highly enriched (47.97%) especially in K farm housing 56 days old turkeys as shown in Figure 1C. In case of Bacteroidetes, this phylum was found the highest in the flock C of M farm housing 63 days old turkeys (M-C-63; 22.38%) followed by K farm having turkeys at 84 days old (K-84; 17.26%).

**Figure 2:**
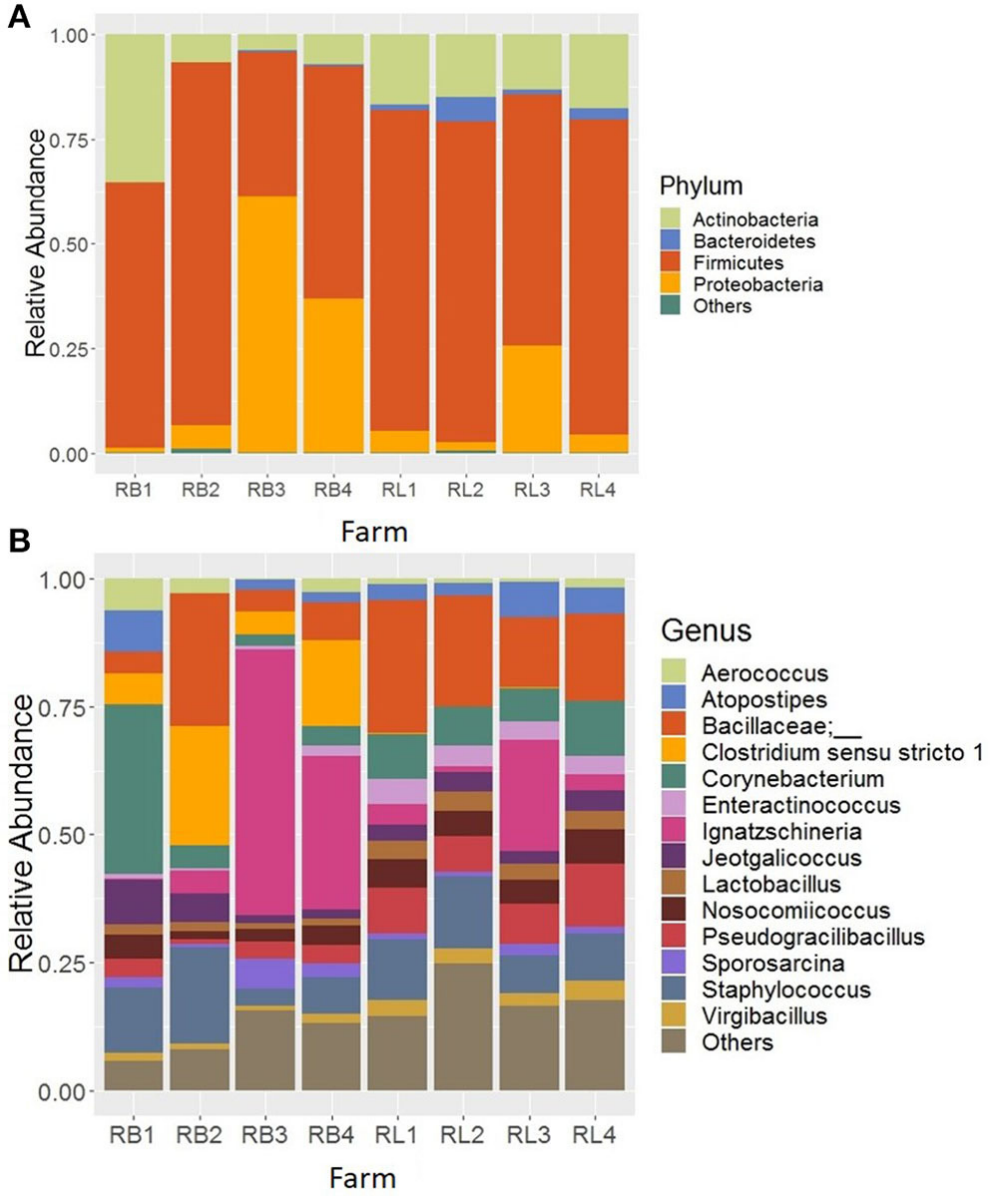
Composition of the litter microbiotas in R farm with incidence of cellulitis at **(A)** phylum and **(B)** genus level. RB and RL represent sponge swab samples collected directly from the birds with cellulitis and boot sponge swab samples collected from the litter surrounding those birds, respectively. “Others” in **(A)** represent the minor phyla whose relantive abundance were <0.1% and in **(B)** the minor genera whose relative abundance were >2.0%.

In the samples from cellulitis-positive farm (R farm), Firmicutes was detected as the predominant phylum (66.06%) followed by Proteobacteria (17.77%), Actinobacteria (14.44%), and Bacteroidetes (1.47%), which constituted 99.97% of the total sequence reads. Although no direct comparisons can be made, the relative abundance of phyla Firmicutes and Proteobacteria were increased, while the relative abundance of phyla Actinobacteria and Bacteroidetes decreased in cellulitis-positive farm samples in comparison to the rest of the farm samples. The distribution of the relative abundance of major four phyla across different samples from R farm is shown in Figure 2A. The phylum Bacteroidetes was significantly reduced in bird swab samples (RB; 0.19%) as compared to the litter swab samples (RL; 2.75%) at *P* < 0.05 (Kruskal-Wallis test). In addition, Proteobacteria was numerically enriched in RB (26.22 vs. 9.31%), whereas Firmicutes (72.15 vs. 59.98%) and Actinobacteria (15.60 vs. 13.28%) were numerically abundant in RL.

### Genus Level Compositions of Litter Bacterial Communities

**Figure 3:**
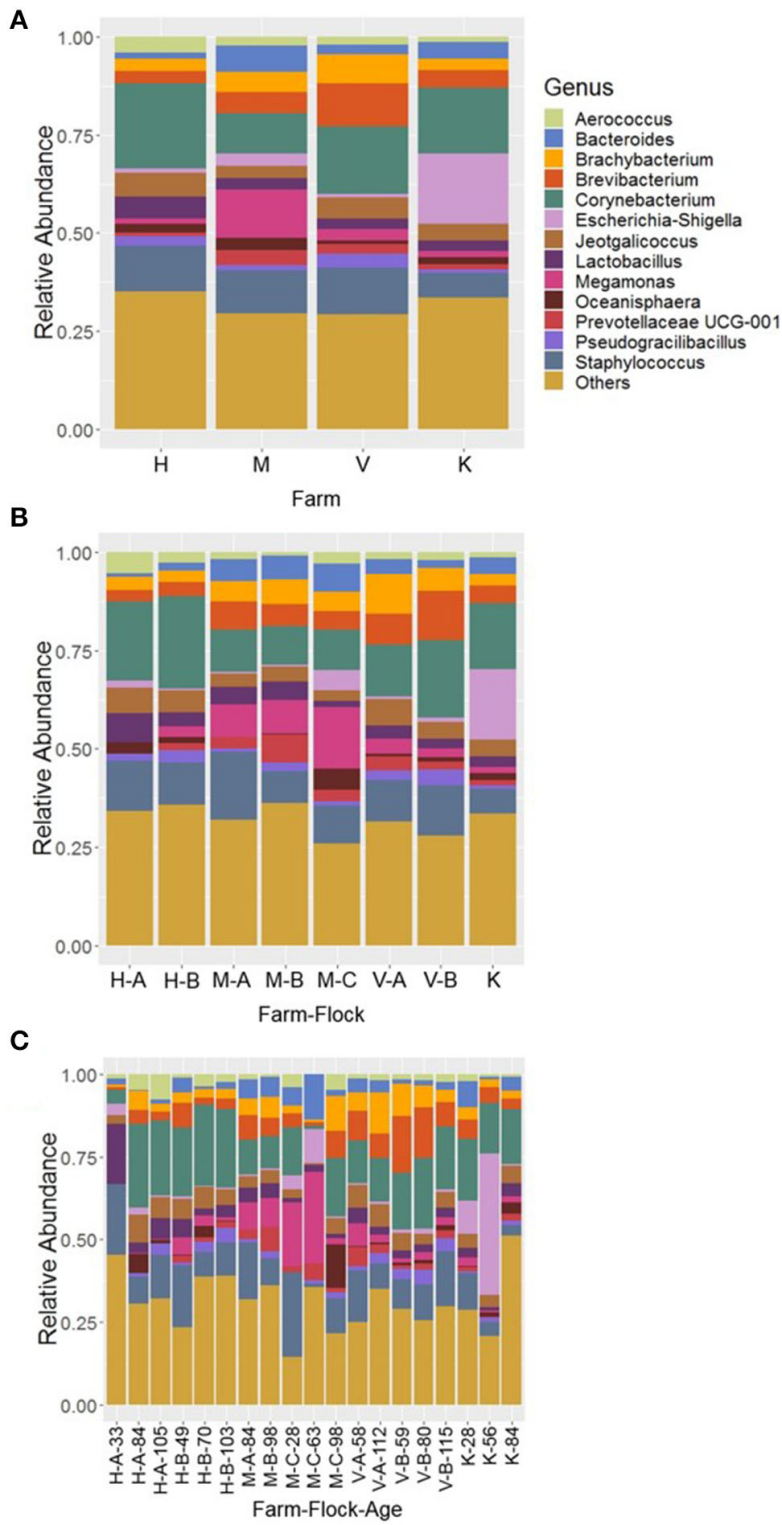
Composition of the litter microbiotas in four different commercial turkey farms of Northwest Arkansas at genus level for different **(A)** farms, **(B)** farm-flocks, and **(C)** farm-flock-ages. “Others” represent the minor genera whose relative abundance were <2.0%.

At genus level, 13 major bacterial genera were identified whose average relative abundance were >2% when summed across all four farm samples excluding R farm. Among these genera, the relative abundance of the genus *Corynebacterium* (16.66%) was found the highest, followed by *Staphylococcus* (11.03%), *Brevibacterium* (6.01%), *Megamonas* (5.13%), *Brachybacterium* (4.83%), *Jeotgalicoccus* (4.76%), *Lactobacillus* (3.72%), *Bacteroides* (3.66%), *Escherichia*-*Shigella* (3.33%), *Aerococcus* (2.62%), Prevotellaceae UCG-001 (2.27%), *Pseudogracibacilibacillus* (2.24%), and *Oceanisphaera* (2.04%). The relative abundance of the major genera across four different farms is shown in Figure 3A. The genus *Corynebacterium* was the predominant genus in H (21.78%) and V (17.30%) farm, however, the genera *Megamonas* (12.39%) and *Escherichia*-*Shigella* (17.79%) were significantly higher in the M and K farm, respectively, at *P* < 0.05. Moreover, the composition of bacterial genera varied not only across the different flocks of the same farm (Figure 3B), but was also affected by ages of birds within the same flock (Figure 3C). For instance, the genus *Megamonas* was highly enriched in flock C of the M Farm rearing turkeys of 28 (M-C-28; 19.02%) and 63 days old (M-C-63; 27.60%), but very lower level of *Megamonas* was detected at the same flock rearing 98 days old (M-C-98; 1.95%) turkeys. Similarly, the genus *Escherichia*-*Shigella* was highly abundant in K farm having the turkeys of 56 days old (42.83%) (Figure 3C). Similarly, the genus Bacteroides was the highest from the flock C of M Farm rearing turkeys of 63 days old (M-C-63; 13.70%). Regarding *Corynebacterium* and *Staphylococcus*, they were present at significant amount throughout all ages and flocks of the farms (Figure 3C), except in the flock C of M Farm rearing turkeys of 63 days old (M-C-63) where they were found at 0.97 and 1.07%, respectively.

The top 14 major genera whose relative abundances were on average >2% when summed across all samples recovered from R farm are shown in Figure 2B. On the contrary to the other farm samples, the samples from this cellulitis-positive farm consisted of unknown genera of the family Bacillaceae (15.05%) and *Ignatzschineria* (14.58%), which were only 1.67 and 0.035% in other farm samples, respectively. Other important genera included *Staphylococcus* (10.60%), *Corynebacterium* (9.65%), *Clostridium* sensu stricto 1 (6.34%), *Pseudogracilibacillus* (5.95%), *Nosocomiicoccus* (4.28%), *Jeotgalicoccus* (3.88%), Atopostipes (3.69%), *Lactobacillus* (2.55%), *Enteractinococcus* (2.54%), *Virgibacillus* (2.20%), *Sporosarcina* (2.09%), and *Aerococcus* (2.06%). Although direct comparisons cannot be made, it seems that different genera were differentially abundant between the cellulitis-positive farm samples (Figure 2B) and the rest of the farm samples (Figure 3A). Moreover, as seen in Figure 2B, there existed differences in the relative abundance of major bacterial genera between RL (litter swabs) and RB (bird swabs) groups. For instance, the genera *Enteractinococcus, Pseudogracilibacillus, Virgibacillus, Nosocomiicoccus*, and *Lactobacillus* were significantly higher in RL group, while the *Clostridium* sensu stricto 1 was significantly higher in RB group (Kruskal-Wallis test, *P* < 0.05).

When all ASVs that belong to the *Clostridium* sensu stricto 1 were compared with *Clostridium septicum* 16S rRNA gene sequence, they showed > 97% similarity. Thus, we believed that the sequence reads of *Clostridium* sensu stricto 1 belong to *C*. *septicum* as *C. septicum* is considered as the primary etiological agent of cellulitis in turkeys (25) and these sequence reads were detected exclusively in the samples from R farm.

### Core Bacterial Genera

The number of core bacterial genera that were present in the 50–100% of all litter swab samples except those from R farm are shown in Supplementary Figure 1. There were 90 core bacterial genera found in 50% of the samples, while only 4 genera (*Staphylococcus, Brevibacterium, Brachybacterium*, and *Lactobacillus*) were found in all samples (Supplementary Figure 1). In addition, 20 core bacterial genera were identified in 95% of the samples, which include *Corynebacterium, Staphylococcus, Jeotgalicoccus, Brevibacterium, Brachybacterium, Lactobacillus, Bacteroides, Pseudogracilibacillus, Aerococcus, Atopostipes, Virgibacillus*, an unknown genus of Lachnospiraceae, *Facklamia, Weissella, Escherichia*-*Shigella, Bifidobacterium, Enterococcus, Phascolarctobacterium, Sellimonas*, and *Subdoligranulum*.

On the contrary, the number of core bacterial genera that were present in the 50–100% of the samples from R farm (with the incidence of cellulitis) are shown in Supplementary Figure 2. As shown in Supplementary Figure 2, 73 core bacterial genera were detected in 50% of samples, whereas 24 genera were present in all 100% samples. These genera include unknown genus of Bacillaceae, *Staphylococcus, Corynebacterium, Pseudogracilibacillus, Nosocomiicoccus, Ignatzschineria, Jeotgalicoccus, Atopostipes, Enteractinococcus, Lactobacillus, Virgibacillus, Sporosarcina, Aerococcus, Weissella, Brevibacterium*, an uncultured genus of Bacillaceae, *Bifidobacterium, Brachybacterium*, an unknown genus of Lachnospiraceae, *Salinicoccus, Subdoligranulum, Blautia, Sellimonas*, and *Romboutsia*.

### Alpha Diversity

**Figure 4:**
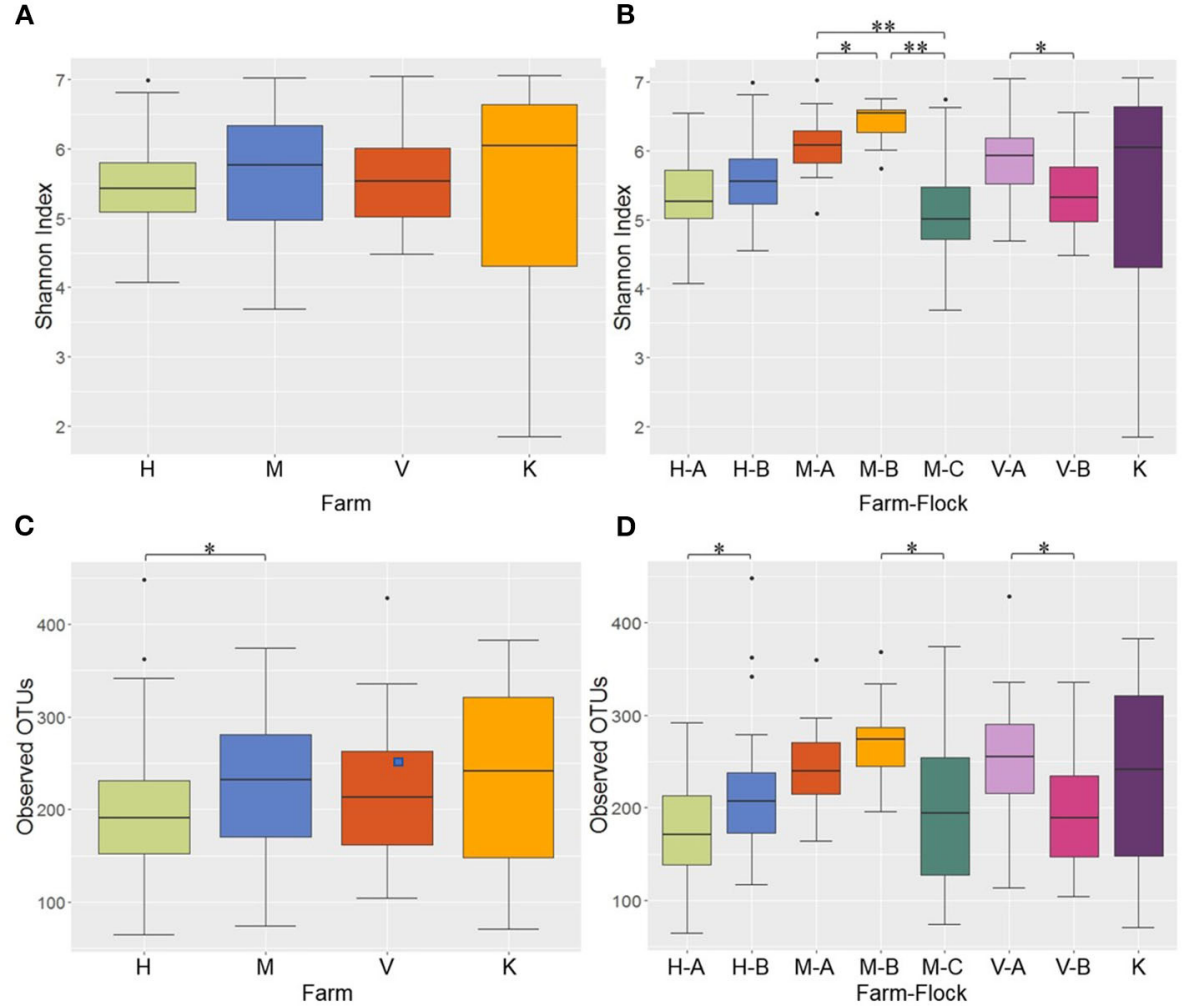
Alpha diversity of the litter microbiotas in four different farms of Northwest Arkansas for different farms and farm-flocks as measured by Shannon Index [**(A,B)**, respectively] and Observed OTUs [**(C,D)**, respectively]. Significant difference is indicated at adjusted *P* (*q*) < 0.05 (*) or < 0.01(**).

Alpha diversity of the microbial communities was measured using Shannon and observed OTUs indices. When the Shannon index was compared among the 4 different farms, no significant difference was observed in alpha diversity (Figure 4A). However, when the Shannon index was compared across different flocks within the same farms, all pairwise comparisons among the 3 flocks (A, B, and C) in M Farm showed significant differences (*P* < 0.05) (Figure 4B). Similarly, the two flocks (A and B) in V Farm showed significant difference in the Shannon index (Figure 4B; adjusted *P* < 0.05). Similar, yet slightly different results were observed with observed OTU index. There was significant difference in alpha diversity between H and M Farms (Figure 4C) (*P* < 0.05). When the flocks within the same farms were compared, significant difference (*P* < 0.05) was observed between the flock A and B in H Farm, between the flock B and C in M Farm, and between the flock A and B in V Farm (Figure 4D).

### Beta Diversity

**Figure 5:**
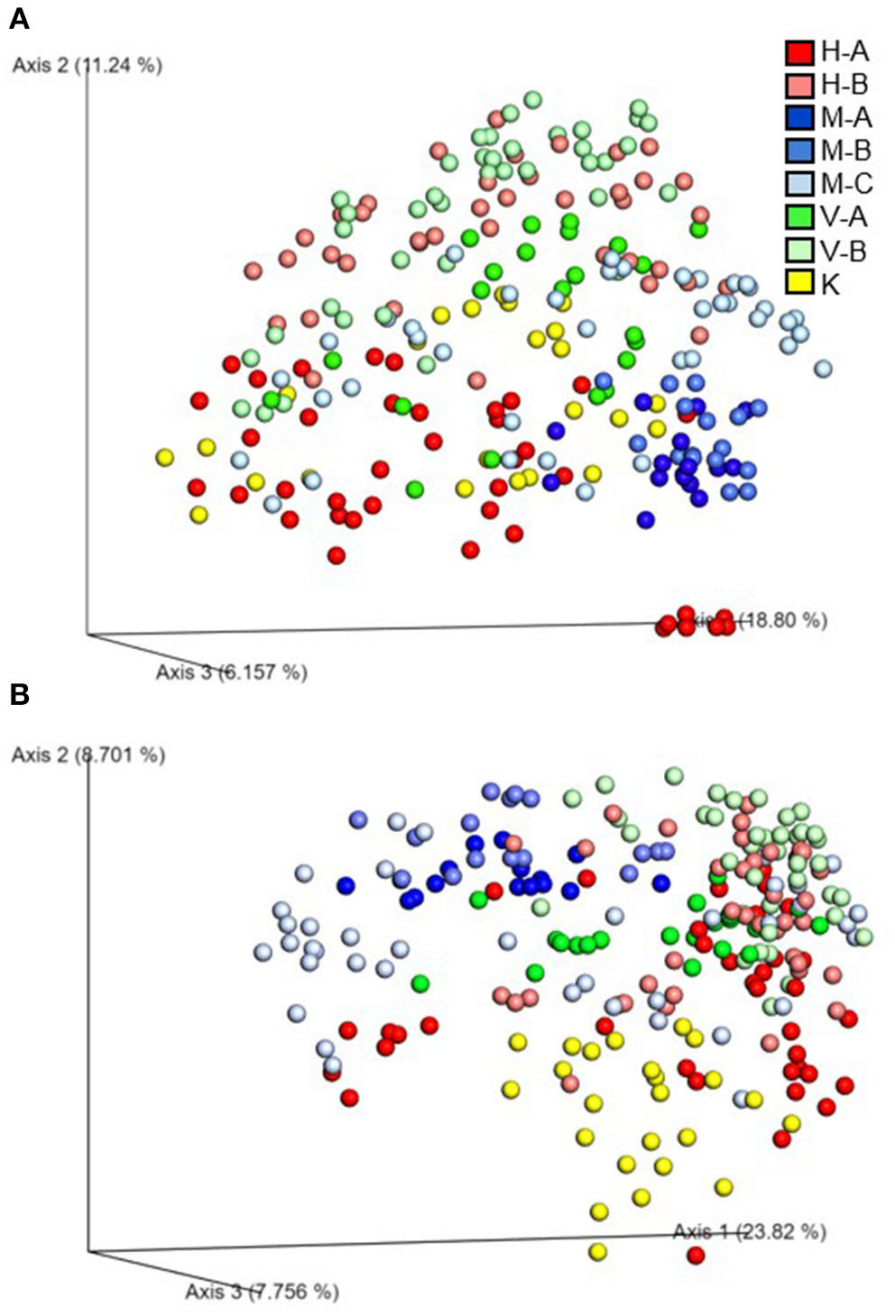
Emperor plot showing beta diversity distances among the different samples from the four farms in Northwest Arknasas as measured by **(A)** unweighted UniFrac distance and **(B)** Bray-Curtis distance indices. A, B, and C represent different flocks.

**Figure 6:**
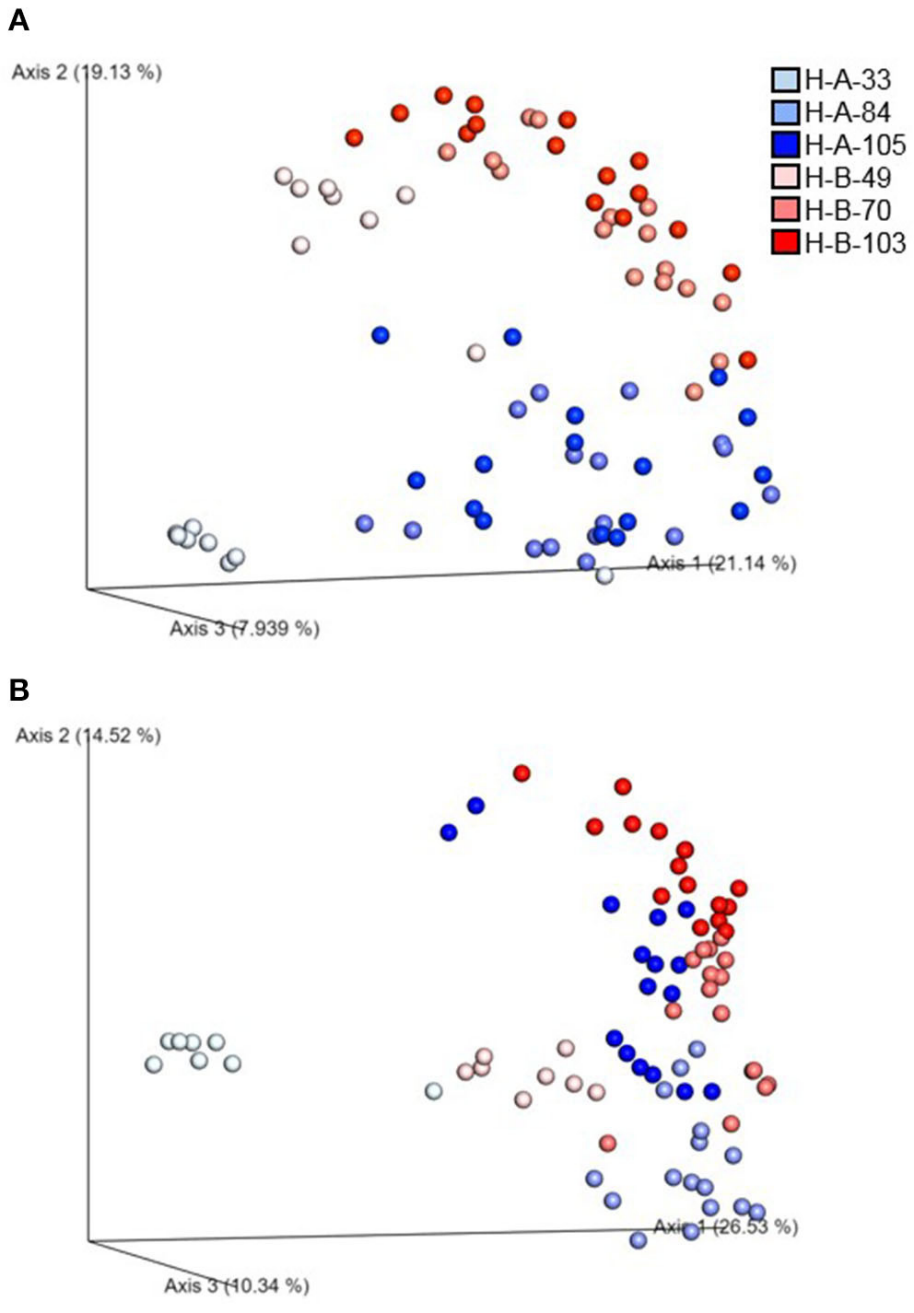
Emperor plot showing beta diversity distances among the different samples in H farm in Northwest Arkansas as measured by **(A)** unweighted UniFrac distance and **(B)** Bray-Curtis distance indices. **(A,B)** represent two different flocks of H farm, whereas the number represents the ages of turkeys when samples were collected.

Beta diversity of the microbial communities was measured by unweighted distance and Bray-Curtis metrics. All pairwise combinations of various flocks from four turkey farms showed significant difference in microbial communities among the groups as indicated by both unweighted distance metric (Figure 5A; adjusted *P* < 0.001) and Bray-Curtis distance metric (Figure 5B; adjusted *P* < 0.01). In addition, within H farm, all possible pairwise comparisons of flocks and ages combinations showed significantly different microbial community structure in terms of both unweighted distance metrics (Figure 6A) and Bray-Curtis (Figure 6B) at adjusted *P* < 0.001.

## Discussion

In the current study, we characterized the microbiotas associated with the litter from five different commercial farms of the Northwest Arkansas, including a farm with positive incidence of cellulitis. To our knowledge, this is the first study that used boot swab samples for comprehensive survey of litter microbiotas in commercial turkey farms. Previously, boot swab was used for detection of *Mycobacterium avium* subsp. paratuberculosis (MAP) in cattle herds (26). By the culture of boot swab samples, they were able to isolate MAP from 90.6% of MAP confirmed cattle herds. We also noticed significant enrichment of *Clostridium* sensu stricto 1 in farm samples with positive incidence of cellulitis (R farm). When the sequences of all ASVs identified as *Clostridium* sensu stricto 1 were compared with *C*. *septicum* 16S rRNA gene sequence, they shared > 97% sequence identity. Furthermore, the results of a nested qPCR assay targetting alpha toxin gene (*csa*) of *C*. *septicum* gave strong amplification signals from the farm samples with incidence of cellulitis (data not shown). Thus, we believe that the sequences that were classified as *Clostridium* sensu stricto 1 belong to *C*. *septicum*, since cellulitis in turkey is considered to be primarily caused by *C*. *septicum* (25). This further suggests that the boot swab samples can serve as an easy and cost-effective technique for the collection of environmental samples for detection of various pathogens as well as characterization of litter microbiotas in poultry farms. Moreover, studies on litter microbiota can reflect the changes in the microbial communities of the poultry as the litter microbial communities correlate with those residing in the poultry hosts (8), which are further affected by the litter types (27).

It was found different flocks with in the same farm contributed to differences in the composition and structures of litter microbial communities, which are further affected by the ages of turkeys. Age as a major driving factor of turkey microbiota was also reported previously (7, 8). Differences in environmental conditions can play a vital role in the initial maturation of turkey microbiota, in addition to the flock types (7). Although the trend is not linear, we noticed the higher abundance of Firmicutes from the flocks rearing younger age of birds, while Actinobacteria and Bacteroidetes were reported higher from the flocks rearing older birds.

Interestingly, the phyla Proteobacteria and Bacteroidetes were highly enriched in the flock C of the M farm with 63 days old turkeys (M-C-63) and K farm housing 56 days old turkeys (K-56), respectively. This was reflected at the genus level by increased abundance of *Escherichia*-*Shigella* and *Bacteroides* in the respective farms. The Proteobacteria is the phylum that contains several pathogenic Increase in relative abundance of Gram-negative genera such as *Escherichia* and *Shigella* is generally considered as the signature of gut dysbiosis (28). Therefore, increase in the relative abundance of the phylum Proteobacteria and the subsequent increase of genera *Escherichia*-*Shigella* in the K farm (K-56) might be indicative of gut dysbiosis of turkeys, though we are lacking any data to support our hypothesis. Another important observation was that the genera *Bacteroides* and *Megamonas* were present the most in the M-C-63 group. The increase in the relative abundance of *Bacteroides* in the particular farm was explained by the highest abundance of the phylum Bacteroidetes in that farm. In addition, the genus *Staphylococcus* was highly reduced in M-C-63 as compared to the other groups. The *Bacteroides* is a genus of Gram- negative bacteria that are well-known for its ability to degrade complex plant carbohydrates and host derived glycan. This group of bacteria can exert beneficial effects toward the hosts' health, maintaining gut homeostasis. However, such effects were found to vary among the studies and due to the strains of *Bacteroides* (29, 30). The increase in the abundance of the genera *Bacteroides* and *Megamonas* might be associated with the reduction of *Staphylococcus* in M-C-63 group. Although *C*. *septicum* is considered as primary etiological agent, *Staphylococcus aureus* was also reported to be associated with cellulitis in turkeys (31). This was further supported by our results from the farm with positive incidence of cellulitis, where the *Staphylococcus* was detected in all samples, suggesting the possible association of *Staphylococcus* in cellulitis of turkeys.

Moreover, only 4 core genera (*Staphylococcus, Brevibacterium, Brachybacterium*, and *Lactobacillus*) were found in all samples of 4 farms excluding those from R farm, whereas 24 core genera were present in all samples from R farm that had cellulitis. The important core genera in cellulitis-positive samples were *Corynebacterium*, an unknown genus of family Bacillaceae, *Clostridium* sensu stricto 1 (> 97% similarity with *C*. *septicum*), and *Ignatzschineria* among others. These genera should be considered while describing the etiopathogenesis of cellulitis in turkeys. The genus *Ignatzschineria* was noticeably enriched in some of the positive samples especially in RB3 (51.97%), RB4 (29.91%), and RL3 (21.70%) as shown in Figure 2B. *Ignatzschineria* is a genus of Gram-negative bacteria that has been associated with necrotizing wounds colonized by maggots (32–34). This group of bacteria are common isolates from the larvae of the parasitic flesh fly (*Wohlfahrtia magnifica*) and two species, *I*. *indica* (32, 34) and *I*. *ureiclastica* (33) were isolated from the bacteremia following maggot's infestation of the wounds in humans. This suggests that if the cellulitis is not properly treated in a timely manner, it might create further complications including septicemia.

In sum, boot swab samples were successfully used to investigate the litter microbial communities of the commercial turkey farms of the Northwest Arkansas. Majority of the microbial taxa identified using boot swabs belong to the microbiota residing in the gut of the poultry, which suggests that the litter microbiota might be used to reflect the microbial changes in the hosts. The composition and diversities of litter microbial communities varied even among the flocks of the same farm, which were further affected by the age of turkeys. The core bacterial genera from samples with cellulitis differed from those for the rest of the farm samples. In addition, several bacterial genera such as *Corynebacterium, Staphylococcus, Ignatzschineria*, and unknown genus of family Bacillaceae that were identified as core members in the cellulitis-positive samples might be correlated with incidence of cellulitis in addition to *C*. *septicum*.

## Data Availability Statement

The datasets presented in this study can be found in online repositories. The names of the repository/repositories and accession number(s) can be found at: https://www.ncbi.nlm.nih.gov/, PRJNA657026.

## Author Contributions

GT-I, BW, and YK conceived and designed the study. BW coordinated the collection of litter boot sponge swab samples and data. BA processed the swab samples, performed the DNA extraction, and library preparation. BA and YK performed all data analysis and wrote the manuscript. YK and TJ supervised the study. All authors provided critical comments and approved the final version of the manuscript.

## Conflict of Interest

BW was employed by Cargill Inc. The remaining authors declare that the research was conducted in the absence of any commercial or financial relationships that could be construed as a potential conflict of interest.

## References

[1] Muir P Li S Lou S Wang D Spakowicz DJ Salichos L The real cost of sequencing: scaling computation to keep pace with data generation. Genome Biol. (2016) 17:1–9. 10.1186/s13059-016-0961-9 27009100 PMC4806511

[2] Jovel J Patterson J Wang W Hotte N O'Keefe S Mitchel T Characterization of the gut microbiome using 16S or shotgun metagenomics. Front Microbiol. (2016) 7:459 10.3389/fmicb.2016.00459 27148170 PMC4837688

[3] Scupham AJ. Succession in the intestinal microbiota of preadolescent turkeys. FEMS Microbiol Ecol. (2007) 60:136–47. 10.1111/j.1574-6941.2006.00245.x 17284250

[4] Scupham AJ. Campylobacter colonization of the turkey intestine in the *context* of microbial community development. Appl Environ Microbiol. (2009) 75:3564–71. 10.1128/AEM.01409-08 19346343 PMC2687274

[5] Scupham AJ Patton TG Bent E Bayles DO. Comparison of the cecal microbiota of domestic and wild turkeys. Microbial Ecol. (2008) 56:322–31. 10.1007/s00248-007-9349-4 18183454

[6] Jami E White BA Mizrahi I. Potential role of the bovine rumen microbiome in modulating milk composition and feed efficiency. PLoS ONE. (2014) 9:e85423. 10.1371/journal.pone.0085423 24465556 PMC3899005

[7] Danzeisen JL Calvert AJ Noll SL McComb B Sherwood JS Logue CM. Succession of the turkey gastrointestinal bacterial microbiome related to weight gain. PeerJ. (2013) 1:e237. 10.7717/peerj.237 24432198 PMC3883494

[8] Danzeisen JL Clayton JB Huang H Knights D McComb B Hayer SS. Temporal relationships exist between cecum, ileum, and litter bacterial microbiomes in a commercial turkey flock, and subtherapeutic penicillin treatment impacts ileum bacterial community establishment. Front Vet Sci. (2015) 2:56. 10.3389/fvets.2015.00056 26664983 PMC4672264

[9] D'Andreano S Bonastre AS Francino O Martí AC Lecchi C Grilli G. Gastrointestinal microbial population of turkey (Meleagris gallopavo) affected by hemorrhagic enteritis virus. Poult Sci. (2017) 96:3550–8. 10.3382/ps/pex139 28938792

[10] Wilkinson TJ Cowan AA Vallin HE Onime LA Oyama LB Cameron SJ. Characterization of the microbiome along the gastrointestinal tract of growing turkeys. Front Microbiol. (2017) 8:1089. 10.3389/fmicb.2017.01089 28690591 PMC5479886

[11] Knudsen BE Bergmark L Munk P Lukjancenko O Priem é A Aarestrup FM Pamp SJ. Impact of sample type and DNA isolation procedure on genomic inference of microbiome composition. Msystems. (2016) 1:e00095–16. 10.1128/mSystems.00095-16 27822556 PMC5080404

[12] Parada AE Needham DM Fuhrman JA. Every base matters: assessing small subunit rRNA primers for marine microbiomes with mock communities, time series and global field samples. Environ Microbiol. (2016) 18:1403–14. 10.1111/1462-2920.13023 26271760

[13] Apprill A McNally S Parsons R Weber L Minor revision to V4 region SSU rRNA 806R gene primer greatly increases detection of SAR11 bacterioplankton. Aquat Microb Ecol. (2015) 75:129–37. 10.3354/ame01753

[14] Thompson LR Sanders JG McDonald D Amir A Ladau J Locey KJ. Earth microbiome project consortium. a communal catalogue reveals Earth's multiscale microbial diversity. Nature. (2017) 551:457–63. 10.1038/nature24621 29088705 PMC6192678

[15] Bolyen E Rideout JR Dillon MR Bokulich NA Abnet C Al-Ghalith GA Reproducible, interactive, scalable and extensible microbiome data science using QIIME 2. Nat Biotechnol. (2019) 37:852–7. 10.1038/s41587-019-0209-9 31341288 PMC7015180

[16] Callahan BJ McMurdie PJ Rosen MJ Han AW Johnson AJA Holmes SP. DADA2: high-resolution sample inference from Illumina amplicon data. Nat Methods. (2016) 13:581–3. 10.1038/nmeth.3869 27214047 PMC4927377

[17] Pedregosa F Varoquaux G Gramfort A Michel V Thirion B Grisel O Scikit-learn: machine learning in Python. J Mach Learn Res. (2011) 12:2825–30. 10.5555/1953048.2078195

[18] Yilmaz P Parfrey LW Yarza P Gerken J Pruesse E Quast C. The SILVA and “All-species Living Tree Project (LTP)” taxonomic frameworks. Nucl Acids Res. (2014) 42:D643–8. 10.1093/nar/gkt1209 24293649 PMC3965112

[19] Quast C Pruesse E Yilmaz P Gerken J Schweer T Yarza P. The SILVA ribosomal RNA gene database project: improved data processing and web-based tools. Nucl. Acids Res. (2013) 41:D590–D596 23193283 10.1093/nar/gks1219 PMC3531112

[20] Shannon CE A mathematical theory of communication. Bell Syst Tech J. (1948) 27:379–423. 10.1002/j.1538-7305.1948.tb01338.x

[21] Lozupone C Lladser ME Knights D Stombaugh J Knight R. UniFrac: an effective distance metric for microbial community comparison. ISME J. (2011) 5:169–72. 10.1038/ismej.2010.133 20827291 PMC3105689

[22] Bray JR Curtis JT An ordination of the upland forest communities of southern Wisconsin. Ecol Monogr. (1957) 27:325–49. 10.2307/1942268

[23] Wickham H Ggplot2: Elegant Graphics for Data Analysis. New York, NY: Springer (2009). 10.1007/978-0-387-98141-3

[24] Anderson MJ A new method for non-parametric multivariate analysis of variance. Austral Ecol. (2001) 26:32–46. 10.1046/j.1442-9993.2001.01070.x

[25] Tellez G Pumford NR Morgan MJ Wolfenden AD Hargis BM. Evidence for *Clostridium* septicum as a primary cause of cellulitis in commercial turkeys. J Vet Diagn Invest. (2009) 21:374–7. 10.1177/104063870902100313 19407093

[26] Eisenberg T Wolter W Lenz M Schlez K Zschöck M. Boot swabs to collect environmental samples from common locations in dairy herds for Mycobacterium avium ssp. paratuberculosis (MAP) detection *J Dairy Res*. (2013) 80:485–9. 10.1017/S002202991300040X 24103506

[27] Cressman MD Yu Z Nelson MC Moeller SJ Lilburn MS Zerby HN. Interrelations between the microbiotas in the litter and in the intestines of commercial broiler chickens. Appl Environ Microbiol. (2010) 76:6572–82. 10.1128/AEM.00180-10 20693454 PMC2950482

[28] Shin NR Whon TW Bae JW. Proteobacteria: microbial signature of dysbiosis in gut microbiota. Trends Biotechnol. (2015) 33:496–503. 10.1016/j.tibtech.2015.06.011 26210164

[29] Wexler AG Goodman AL. An insider's perspective: bacteroides as a window into the microbiome. Nat Microbiol. (2017) 2:17026. 10.1038/nmicrobiol.2017.26 28440278 PMC5679392

[30] Janssens Y Nielandt J Bronselaer A Debunne N Verbeke F Wynendaele E. Disbiome database: linking the microbiome to disease. BMC Microbiol. (2018) 18:50. 10.1186/s12866-018-1197-5 29866037 PMC5987391

[31] Gornatti-Churria CD Crispo M Shivaprasad HL Uzal FA. Gangrenous dermatitis in chickens and turkeys. J Vet Diagn Invest. (2018) 30:188–96. 10.1177/1040638717742435 29145799 PMC6505868

[32] Barker HS Snyder JW Hicks AB Yanoviak SP Southern P Dhakal BK. First case reports of Ignatzschineria (Schineria) indica associated with myiasis. J Clin Microbiol. (2014) 52:4432–4. 10.1128/JCM.02183-14 25297331 PMC4313336

[33] Le Brun C Gombert M Robert S Mercier E Lanotte P Association of necrotizing wounds colonized by maggots with ignatzschineria–associated septicemia. Emerg Infect Dis. (2015) 21:1881–3. 10.3201/eid2110.150748 26402740 PMC4593450

[34] Muse H Jenkins RL Oliver MB Kim S Grantier RL Malhotra BK. A case of Ignatzschineria indica bacteremia following maggot colonization. Case Rep Infect Dis. (2017) 2017:3698124. 10.1155/2017/3698124 29230335 PMC5688256

