# Supplementary material for: Comprehensive Survey of the Litter Bacterial Communities in Commercial Turkey Farms
Source: Front Vet Sci. 2020 Dec 4;7:596933. doi: 10.3389/fvets.2020.596933 (PMC7746545; doi:10.3389/fvets.2020.596933)
Supplement: Supplementary file 1 [file Data_Sheet_1.docx]

**Supplementary Materials**

Comprehensive survey of the litter bacterial communities

in commercial turkey farms

Bishnu Adhikari^1^, Guillermo Tellez-Isaias^1^, Tieshan Jiang^1^, Brian Wooming^2^, and Young Min Kwon^1,3*^

^1^Department of Poultry Science, University of Arkansas, Fayetteville, AR 72701,

^2^Cargill, Cargill, Inc., Minneapolis, MN 55440,

^3^Cell and Molecular Biology Program, University of Arkansas, Fayetteville, AR 72701


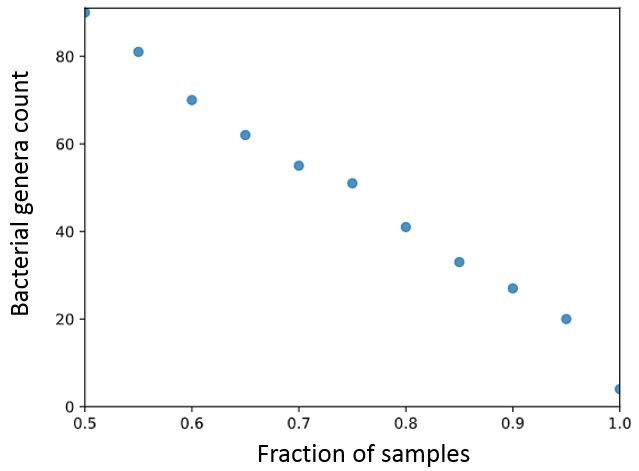


**Figure S1.** The number of core bacterial genera identified from the four different farms of turkeys (H, M, V, and K) and the fraction of samples from which they were recovered.


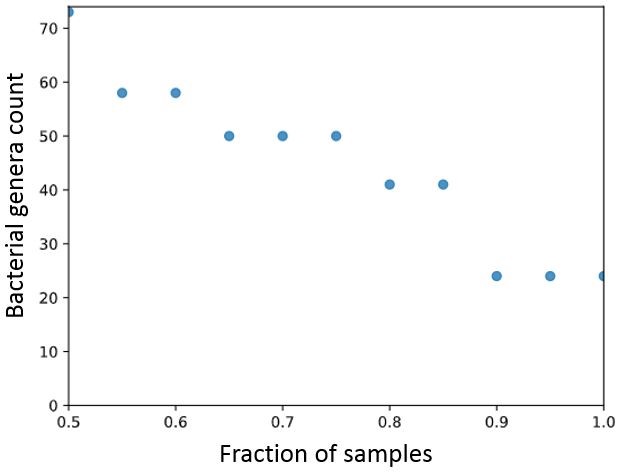


**Figure S2.** The number of core bacterial genera identified from R farm that had an incidence of cellulitis and the fraction of samples from which they were recovered.
